# Nano-indentation study of dislocation evolution in GaN-based laser diodes

**DOI:** 10.1186/s11671-024-03983-0

**Published:** 2024-03-07

**Authors:** Jingjing Chen, Xujun Su, Guobing Wang, Mutong Niu, Xinran Li, Ke Xu

**Affiliations:** 1 Suzhou Institute of Nano-tech and Nano-bionics, CAS, Ruoshui Road 398, Suzhou Industrial Park, Suzhou, 215123 China; 2 Shenyang National Laboratory for Materials Science, Jiangsu Institute of Advanced Semiconductors, Suzhou, 215123 China

**Keywords:** GaN, Dislocation slip system, AlGaN, InGaN

## Abstract

The slip systems and motion behavior of dislocations induced by nano-indentation technique in GaN-based LDs were investigated. Dislocations with burgers vector of b = 1/3 <11$\overline{2}$3> were introduced on either {11$\overline{2}$2} <11$\overline{2}$3>, or {1$\overline{1}$01} <11$\overline{2}$3> pyramidal slip systems in the upper p-GaN layer. Besides, {0001} <11$\overline{2}$0> basal slip system was also activated. The AlGaN/InGaN multi-layers in device can provide mismatch stresses to prevent dislocations from slipping through. It was observed that the density of dislocations induced by the indenter significantly decreased from the upper to the lower regions of the multi-layers. The **a** + **c** dislocations on pyramidal slip planes were mostly blocked by the strained layers.

## Introduction

In recent years, III-nitrides have attracted wide attention due to their excellent properties, including a wide direct band gap, high breakdown electric field, and superior electron transport capabilities [1, 2]. To improve the performance of the GaN-based devices, extensive efforts have been devoted to investigating the influence of various defects on the optical and electrical properties [3–5]. The high density threading dislocations (TDs) can lead to vacancy formation, which compensates for carriers and acts as non-radiative recombination centers in devices [6]. The dislocations in III-nitrides are primarily induced by the mismatch of lattice constants and thermal expansion coefficients between the foreign substrate and the epitaxial film. Additionally, mismatches also occur in multilayer membranes such as heterojunctions or quantum wells, where the element composition varies between the adjacent layers and introduces strain into the system. The misfit dislocations will initiate from the surface and slip into the epitaxial layer to relax the misfit strain when the epitaxial layers exceed a critical thickness [7–9]. The strain relaxation and mechanical deformations in hexagonal lattice systems are related to the dislocation slip systems, which have been widely analyzed [10–12]. Generally, previous studies have analyzed the stress relaxation process by directly observing the dislocation state at the multilayer interface after the growth process, which is easily affected by different growth conditions and high TDs in the substrate.

In recent years, Nano-indentation (NI) has been widely used as an effective method for measuring the formation and movement process of dislocations in GaN and AlN. The dislocations induced by the indenter preferentially slip along {11$\overline{2}$2} <11$\overline{2}$3> or {1$\overline{1}$01} <11$\overline{2}$3> pyramidal slip systems [13, 14]. Compared to the growth dislocation, the dislocations created by NI are more localized and relatively pure characteristics without point defects, which facilitate the investigation of dislocation multiplication and movement mechanisms [15, 16]. As for GaN-based devices with complex epitaxial structures, the dislocations inside are induced by material mismatches or external damage [17–19]. When the device is under high light power, the presence of numerous surface or defect states leads to rapid heating on the cavity surface due to the absorption of a large number of photons. Local transient thermal effects may occur on the cavity surface [20]. During this process, dislocations generated and propagate within the device, potentially extending down to the active region of the quantum wells. Consequently, permanent damage occurs in the device. In this letter, the NI technique was used to artificially induce dislocations in a GaN-based LD with AlGaN/InGaN/multi-layers for investigating their multiplication and motion behavior in devices. The **a** + **c** dislocations were found to slip on {11$\overline{2}$2} and {1$\overline{1}$01} pyramidal planes, while the **c** dislocations slipped on {11$\overline{2}$0} <0001> cylindrical slip system in the device, most of which were ultimately blocked by the stressed multi-layers. These results will enhance our understanding of stress relaxation mechanisms in AlGaN/InGaN multi-layers and failure mechanisms under operating condition.

## Experimental

**Fig. 1:**
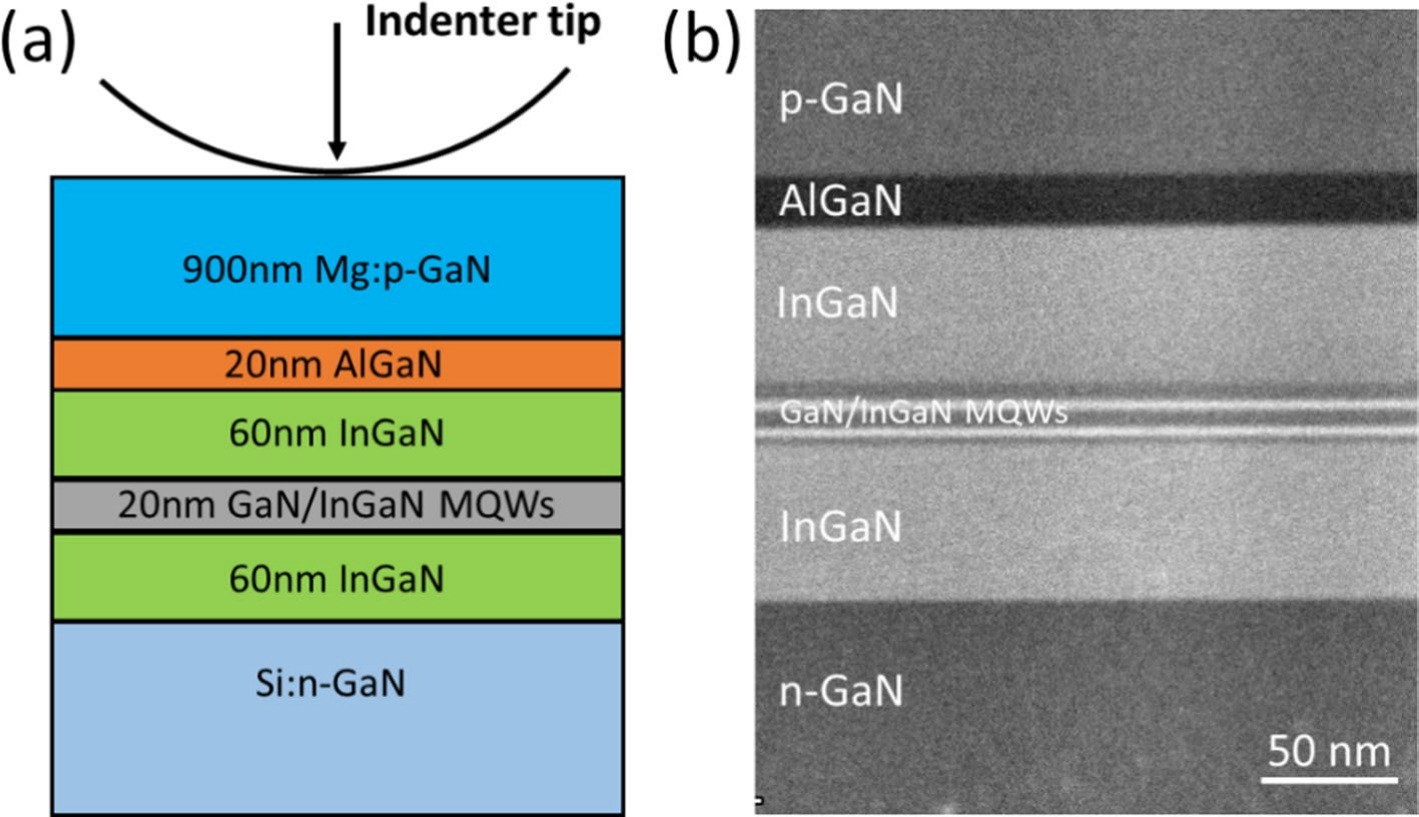
**a** Schematic diagram and **b** STEM image of GaN-based LD structure

The GaN-based LD device used in this study was homo-epitaxial grown on a free-standing GaN substrate by metalorganic chemical vapor deposition (MOCVD). The density of the threading dislocation in device is about ${ }10^{8} /{\text{cm}}^{2}$. The epilayers structure comprises a p-GaN contact layer, an Al_0.12_Ga_0.88_N electron barrier layer, two In_0.02_Ga_0.98_N waveguide layers, GaN/In_0.06_Ga_0.94_N multiple quantum well, and an n-contact GaN layer, as shown in Fig. 1. Nano-indentation experiments were conducted using a Nano-indenter G200 system with a diamond conical-shaped indenter tip with a curvature radius of 5 μm. The strain rate was set at 0.05 nm/s during nano-indentation tests, and the maximum indentation depth was about 215 nm. Two TEM samples (A and B) were prepared by focused ion beam (FIB) milling at the indentation position along zone axis (ZA) of [1$\overline{1}$00] and [11$\overline{2}$0], respectively. The defects induced by the indenter were investigated by transmission electron microscopy (TEM) using a FEI Talos instrument operated at 200 kV.

## Results and discussion

**Fig. 2:**
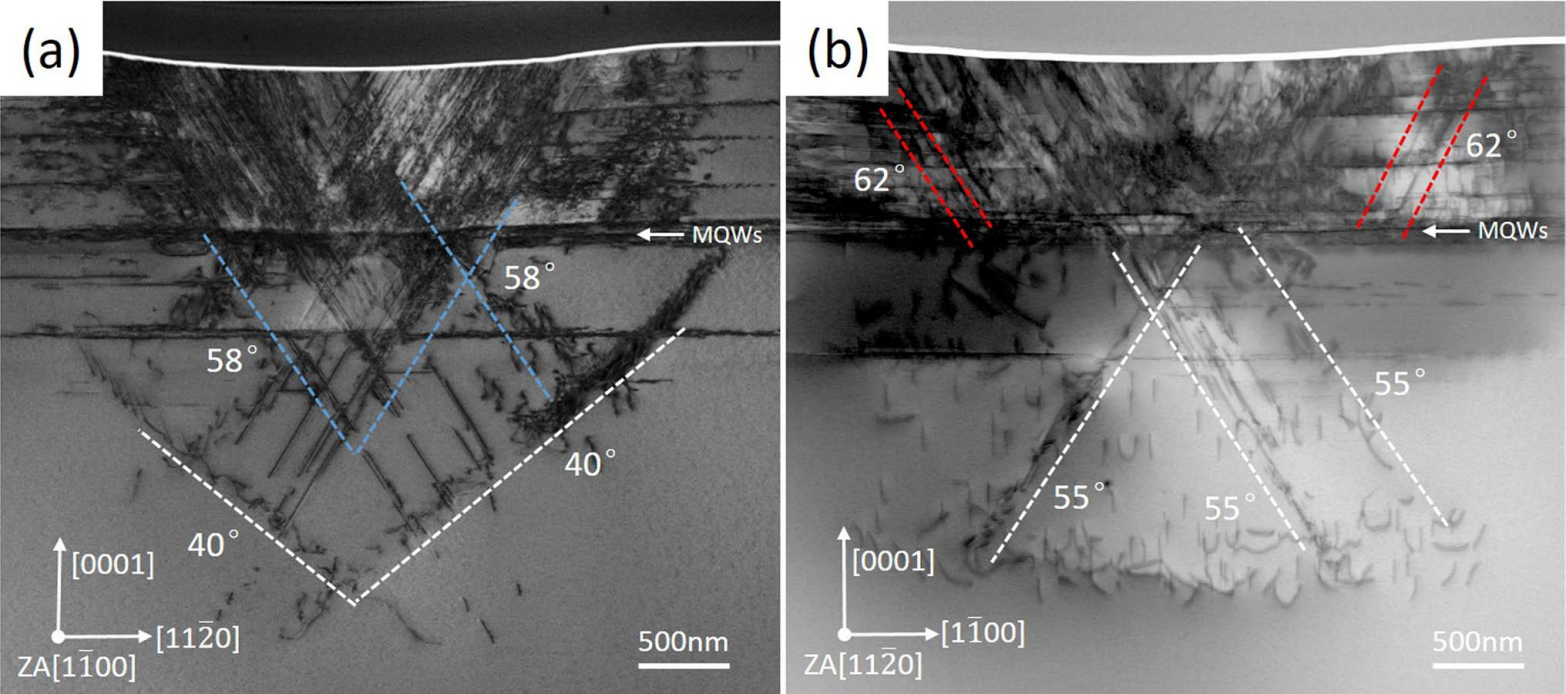
STEM-HAADF images of **a** samples A (ZA [1$\overline{1}$00]) and **b** sample B (ZA [11$\overline{2}$0]). The dashed lines indicate the dislocation slip planes, the angles between these lines and [11$\overline{2}$0] or [1$\overline{1}$00] were also marked

The STEM-HAADF images in Fig. 2a, b were taken along the zone axis of [1$\overline{1}$00] and [11$\overline{2}$0], respectively. The extreme stress caused by the indenter results in a large number of dislocations on the device. In Fig. 2a, several inclined dislocation bands are at angles of 58° or 40° to [11$\overline{2}$0] direction. In Fig. 2b, the inclined dislocations bands below the quantum well symmetrically distributed and at an angle of 55° to [1$\overline{1}$00] direction, and the dislocations bands marked by red dotted lines in all at an angle of about 62° to the [1$\overline{1}$00] direction. Table 1 calculates the angles for three pyramidal slip planes in GaN. Among which, dislocations on {11$\overline{2}$2} and {1$\overline{1}$01} slip planes can form an angle of 58.4° to [11$\overline{2}$0] direction when project to {1$\overline{1}$00} plane, only dislocations on {11$\overline{2}$2} slip planes can form an angle of 54.6° to [1$\overline{1}$00] direction when project to {11$\overline{2}$0} plane. Furthermore, it is noteworthy that only plane {1$\overline{1}$01} exhibits an angle of 62.0° to [1$\overline{1}$00] direction upon projecting to {11$\overline{2}$0} plane. Based upon above analysis, the following conclusions can be drawn: the pyramidal dislocation slip planes present in Fig. 2 are {11$\overline{2}$2} and {1$\overline{1}$01}.

**Table 1:** The angles for the three pyramidal slip planes in GaN

Pyramidal slip plane	The angle between the intersection line of the slip plane and (1$\overline{1}$00) and [11$\overline{2}$0] (°)	The angle between the intersection line of the slip plane and (11$\overline{2}$0) and [1$\overline{1}$00] (°)
{1$\overline{1}$01}	58.4	43.2
	0	62.0
{11$\overline{2}$2}	58.4	54.6
	39.0	0
{1$\overline{1}$02}	39.0	43.2
	0	25

**Fig. 3:**
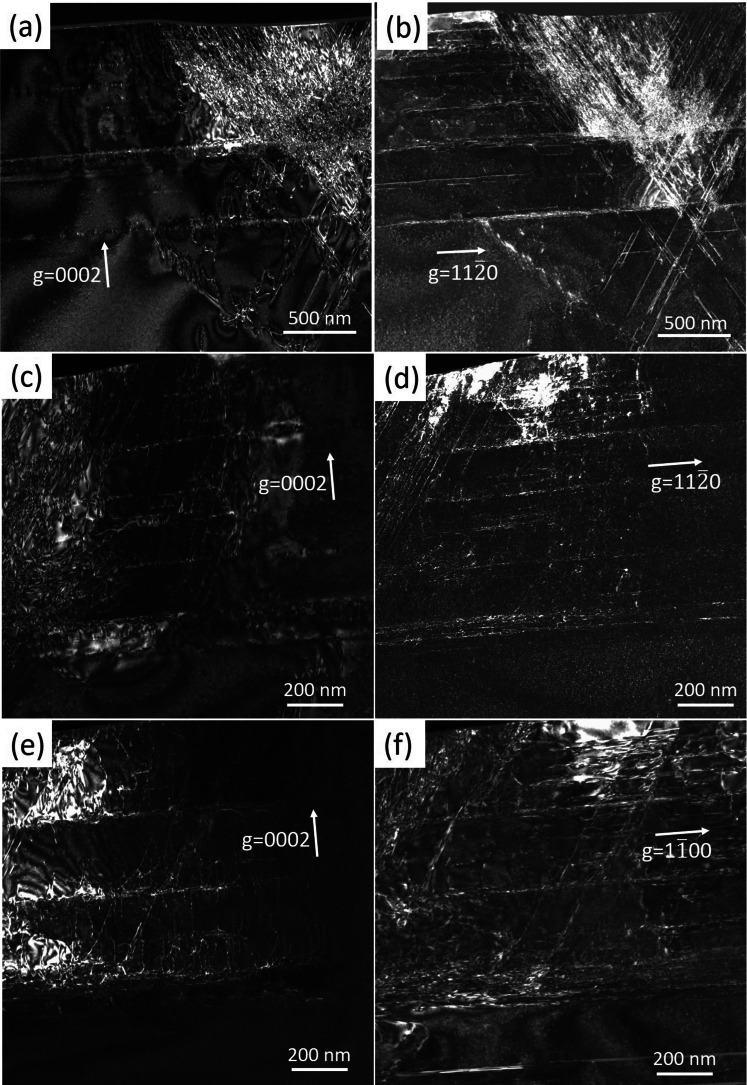
TEM-WBDF images of sample A and sample B. **a**, **c**, **e** were taken with g = 0002 diffraction condition. **b**, **d** were taken with g = 11$\overline{2}$0 diffraction conditions, **f** was taken with g = 1$\overline{1}$00 diffraction condition

The Burgers vectors of dislocations induced by the indenter were investigated by TEM weak-beam dark field (WBDF) images in Fig. 3. Sample A was along zone axis of [1$\overline{1}$00] and characterized by diffraction vectors of **g** = [0002] and **g** = [11$\overline{2}$0], respectively. Sample B was along zone axis of [1$1\overline{2}$0] and characterized by diffraction vectors of **g** = 0002 and **g** = 1$\overline{1}$00, respectively. In Fig. 3a, b, the horizontal dislocations bands located in (0001) slip planes remain invisible under the **g** = 0002 diffraction condition, and observable under the **g** = 11$\overline{2}$0 diffraction condition. It means that these dislocations have Burgers vectors of **b** = 1/3 <11$\overline{2}$0>. The inclined dislocations bands marked by white and blue dashed lines in Fig. 2a are observable under both the **g** = 0002 and **g** = 11$\overline{2}$0 diffraction conditions as shown in Fig. 3a, b, which suggests that the Burgers vectors of these dislocations are 1/3 <11$\overline{2}$3>. The inclined dislocations bands marked by red dashed lines in Fig. 2b are observable under both **g** = 0002 and **g** = 01$\overline{1}$0 diffraction conditions as shown in Fig. 3e, f, which also means that these dislocations have Burgers vectors of **b** = 1/3 <11$\overline{2}$3>. Therefore, the pyramidal slip systems activated by the indenter in GaN-based LD device are {11$\overline{2}$2} <11$\overline{2}$3> and {1$\overline{1}$01} <11$\overline{2}$3>. Based on the distances between the inclined dislocations bands and the indentation point, the activated sequence of the slip systems discussed above can be deduced. The slip system {11$\overline{2}$2} <11$\overline{2}$3> was initially activated in GaN top layer, when the indenter depth increased, slip system {1$\overline{1}$01} <11$\overline{2}$3> was activated then. Caldas et al. have calculated the Schmid factor of conventional slip systems in GaN, only {1$\overline{1}$01} <11$\overline{2}$3>, {11$\overline{2}$2} <11$\overline{2}$3>, and {1$\overline{1}$02} <1$\overline{1}$01> exhibit non-zero values when the external stress is along [000$\overline{1}$] direction [13]. The Peierls–Nabarro critical shear stress $\tau_{c}$ have also been investigated for the conventional slip systems in GaN. The results show that the slip system with the lowest $\tau_{c}$ is {11$\overline{2}$2} <11$\overline{2}$3>, and followed by {1$\overline{1}$01} <11$\overline{2}$3>, which is verified by the inference we drawn.

There are some dislocations parallel to the [0001] direction in Fig. 2b, and these dislocations are visible in g = 0002 diffraction condition but disappear in g = 1$\overline{1}$00, as shown in Fig. 3e, f. These dislocations may have Burgers vectors of **b** =  <0001> or **b** = 1/3 <11$\overline{2}$3>. In Fig. 3b, d, these dislocations were also observed at a similar distance from the center of the indentation. However, the difference was that the orientation of the dislocation lines deviated by approximately 30° from the [0001] direction, indicating that they were located in the pyramidal slip systems rather than in a cylinder. After being projected onto the (11$\overline{2}$0) plane, the dislocation lines are parallel to the [0001] direction. From this, we can infer that the slip plane of dislocations should be {11$\overline{2}$2}, and the Burgers vector is b = 1/3 <11$\overline{2}$3>.

**Fig. 4:**
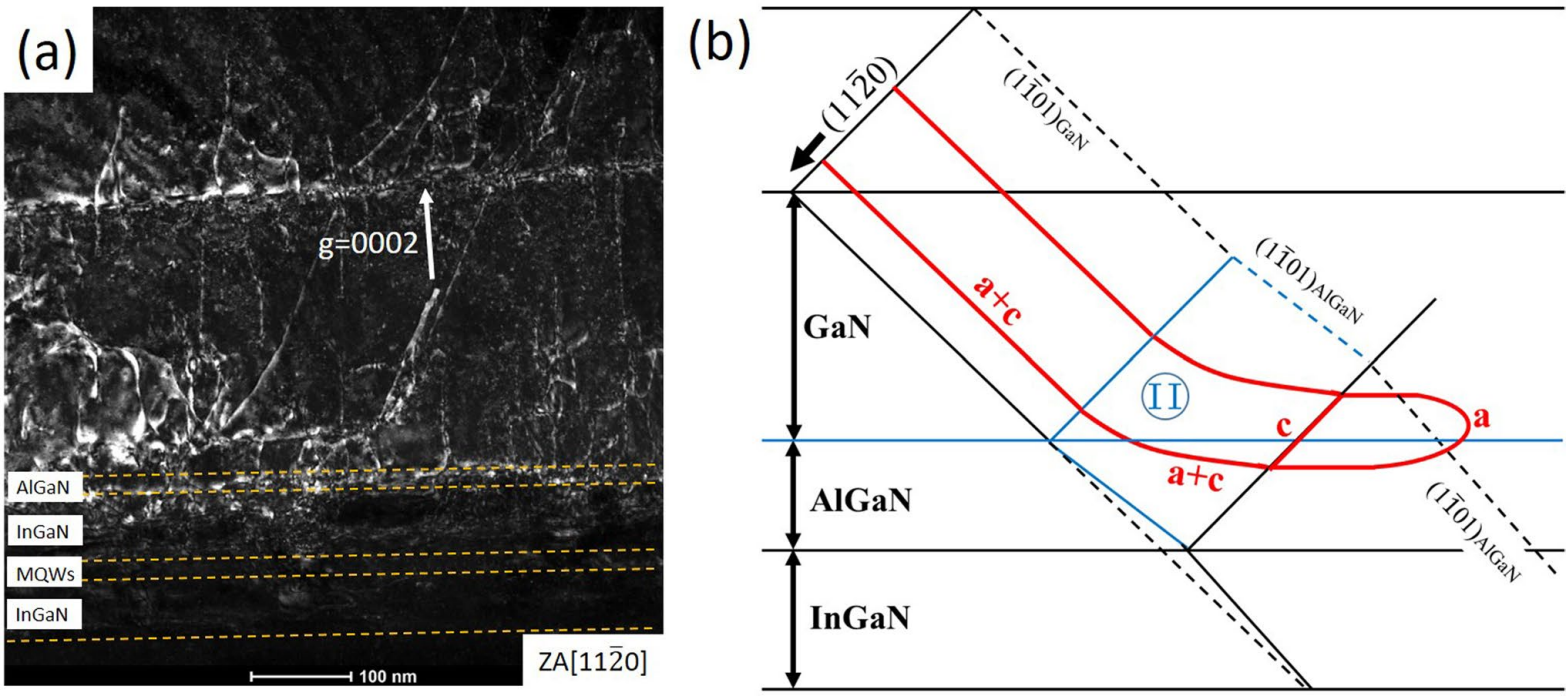
**a** WBDF images with **g** = 0002 around the AlGaN/InGaN multi-layers; **b** The schematic of the motion process of dislocations on pyramidal slip planes in AlGaN/InGaN multi-layers

The dislocation density differs significantly above and below the AlGaN/InGaN multi-layers. Many **a** + **c** dislocations with a Burgers vector of **b** = 1/3 <11$\overline{2}$3> on {11$\overline{2}$2} or {1$\overline{1}$01} slip planes, are blocked by the multi-layers, as shown in Fig. 4a. This phenomenon is more pronounced away from the indentation core. These dislocations on pyramidal planes gradually change their orientation upon encountering the AlGaN layer, with most of them even becoming fully horizontal. This indicates that the AlGaN and InGaN layers play a crucial role in facilitating this motion process. The electron barrier layers (EBL) in GaN-based devices, typically composed of AlGaN materials, confine electrons within the active region to prevent leakage and mitigate the performance degradation of devices [21]. Additionally, AlGaN materials are known to be used in LED and LDs for stress relaxation and prevention of dislocation elongation [10, 22]. Due to the larger lattice constants of GaN crystal (a = 0.3189 nm, c = 0.5185 nm) compared to AlN (a = 0.3112 nm, c = 0.4982 nm), high Al-component AlGaN can induce dislocations bending by providing tensile stress. The lattice constants of InN are greater than those of both GaN and AlN, resulting in dislocations experiencing higher compressive stress as they migrate towards the InGaN layer. When the dislocations with Burgers vectors of **b** = 1/3 <11$\overline{2}$3> propagate from the p-GaN layer to the strained layers, their resistance to slip along the <11$\overline{2}$3> direction is enhanced. Once the stress equilibrium is achieved, these dislocations will stop slipping in their original direction. These **a** + **c** dislocations may undergo bending within the pyramidal plane until they align parallel to the (0001) plane, resulting in mismatched dislocations. There is also another possibility, as demonstrated by Yan et al. [23]. The mismatch of lattice constants results in the accumulation of significant stress within the membrane, which generates shear stress on the basal plane, and potentially leads to the decomposition of **a** + **c** dislocations and cross-sliding along the basal plane. The decomposition process is illustrated in Fig. 4b, and the dissociation equation is

$$\frac{1}{3}\left[ {11\overline{2}3} \right] \to { }\frac{1}{3}\left[ {11\overline{2}0} \right] + \left[ {0001} \right]$$

where the screw component with Burgers vector of 1/3 [11$\overline{2}$0] is capable of cross-slipping, the remaining [0001] edge component is pinned at the interface to form sub-rod dislocation and thus cannot continue to slip. The dissociation is feasible based on the Frank energy criterion, as the total dislocation energy remains constant. However, in Fig. 3d, f, the **a** dislocations in the AlGaN or InGaN layers may also originate from the activation of basal slip systems. The high density dislocations in the stressed layers make it difficult to clearly distinguish the dislocation lines. Therefore, our data is insufficient to determine whether the **a** + **c** dislocations undergo decomposition in the strained layers or bend parallel to (0001) plane. The relaxation mechanism of **a** + **c** dislocations in the strained layers needs further research.

## Conclusions

In summary, nano-indentation experiments were conducted on a GaN-based LD device using a diamond conical-shaped indenter tip with a curvature radius of 5 μm. The activated slip systems in the device were {11$\overline{2}$2} <11$\overline{2}$3>, {1$\overline{1}$01} <11$\overline{2}$3>, and {11$\overline{2}$0} <0001>. The dislocation density induced by the indenter varied greatly in the upper and lower regions of the AlGaN/InGaN multi-layers. According to the TEM images, the dislocations with Burgers vector of **b** = 1/3 <11$\overline{2}$3> on {1$\overline{1}$01} and {11$\overline{2}$2} pyramidal slip planes also stopped slipping in their original direction, and may have undergone decomposition or bend parallel to (0001) plane when passing through the strained layers, which is difficult to determine due to experimental limitations. Further research is needed on the relaxation mechanism of **a** + **c** dislocations in the strained layers.

## Data Availability

The data are available from the corresponding author on reasonable request.
